# Lactate dehydrogenase and susceptibility to deterioration of mild COVID-19 patients: a multicenter nested case-control study

**DOI:** 10.1186/s12916-020-01633-7

**Published:** 2020-06-03

**Authors:** Jichan Shi, Yang Li, Xian Zhou, Qiran Zhang, Xinchun Ye, Zhengxing Wu, Xiangao Jiang, Hongying Yu, Lingyun Shao, Jing-Wen Ai, Haocheng Zhang, Bin Xu, Feng Sun, Wenhong Zhang

**Affiliations:** 1 Department of Infectious Diseases, Wenzhou Central Hospital, Wenzhou, 325000 China; 2 Departments of Infectious Diseases, Huashan Hospital, Fudan University, Shanghai, 200040 China; 3 Department of Infectious Disease, The First People’s Hospital of Huaihua, Huaihua, 418000 China; 4 National Clinical Research Center for Aging and Medicine, Huashan Hospital, State Key Laboratory of Genetic Engineering, School of Life Science, Key Laboratory of Medical Molecular Virology (MOE/MOH) and Institutes of Biomedical Sciences, Shanghai Medical College, Fudan University, Shanghai, 200040 China

**Keywords:** COVID-19, Severe pneumonia, Lactate dehydrogenase, SARS-CoV-2

## Abstract

**Background:**

Coronavirus disease 2019 (COVID-19) has infected more than 4 million people within 4 months. There is an urgent need to properly identify high-risk cases that are more likely to deteriorate even if they present mild diseases on admission.

**Methods:**

A multicenter nested case-control study was conducted in four designated hospitals in China enrolling confirmed COVID-19 patients who were mild on admission. Baseline clinical characteristics were compared between patients with stable mild illness (stable mild group) and those who deteriorated from mild to severe illness (progression group).

**Results:**

From Jan 17, 2020, to Feb 1, 2020, 85 confirmed COVID-19 patients were enrolled, including 16 in the progression group and 69 in the stable mild group. Compared to stable mild group (*n* = 69), patients in the progression group (*n* = 16) were more likely to be older, male, presented with dyspnea, with hypertension, and with higher levels of lactase dehydrogenase and c-reactive protein. In multivariate logistic regression analysis, advanced age (odds ratio [OR], 1.012; 95% confidence interval [CI], 1.020–1.166; *P* = 0.011) and the higher level of lactase dehydrogenase (OR, 1.012; 95% CI, 1.001–1.024; *P* = 0.038) were independently associated with exacerbation in mild COVID-19 patients.

**Conclusion:**

Advanced age and high LDH level are independent risk factors for exacerbation in mild COVID-19 patients. Among the mild patients, clinicians should pay more attention to the elderly patients or those with high LDH levels.

## Background

Coronavirus diseases 2019 (COVID-19) is now officially a pandemic [1]. As of April 1, 2020, the total number of confirmed COVID-19 cases has surpassed 820,000 cases [2]. Research of clinical characteristics of COVID-19 patients began at January, leading by Huang and his colleagues [3]. They reported that more than half of COVID-19 patients developed dyspnea at 8 days following the initial onset of illness, while the onset of acute respiratory distress syndrome (ARDS) had a median day of 9 days, just 1 day more than the onset of dyspnea, which may indicate a rapid diseases progression. Despite the fact that COVID-19 patients have mild symptoms and signs in their early stage, about 8–30% of patients would eventually develop severe illness. The 28-day mortality rate of critically ill patients is over 60% [4]. By far, disease progression in COVID-19 seems to be unpredictable due to our limited understanding of the natural history of the disease. There is an urgent need to properly identify high-risk cases that are more likely to deteriorate even if they present mild diseases on admission.

In light of these uncertainties, we enrolled patients who were evaluated as mild COVID-19 on admission from a prospective cohort in four hospitals. Some of the patients deteriorated to severe diseases. We then compared the baseline characteristics between the stable mild group and progression group, aiming to assess the potential markers to predict whether the disease will progress or not.

## Methods

### Study design and participants

This was a multicenter nested case-control study involving four designated hospitals in China for the treatment of COVID-19 patients. These four hospitals were located in Wenzhou, Wuhan, Huaihua, and Shanghai. The study was approved by the Ethics Committees of Huashan Hospital, Fudan University. All patients who participated in the study gave informed consent.

**Fig. 1:**
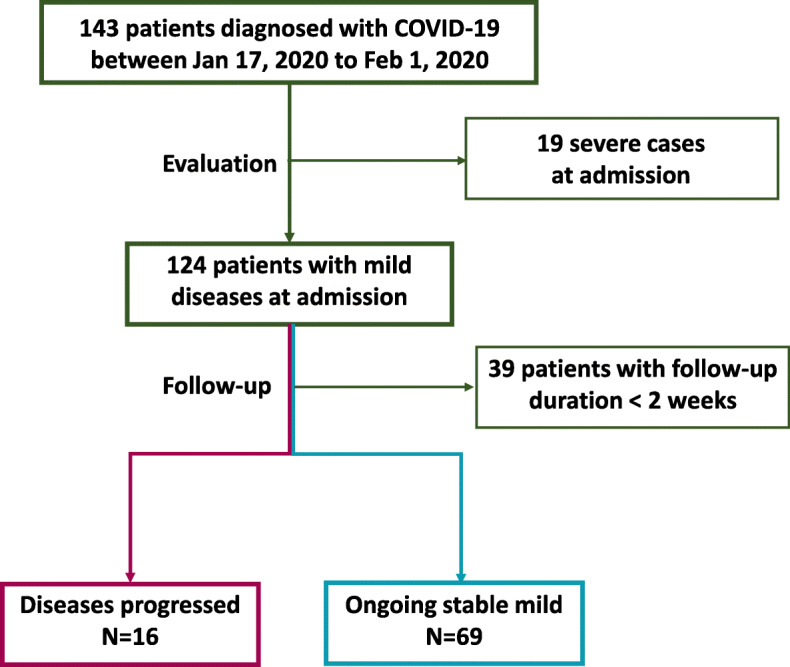
The flow chart of this study

From Jan 17, 2020, to Feb 1, 2020, we enrolled all 143 patients with confirmed COVID-19 according to World Health Organization (WHO) interim guidance [5] in the 4 participated hospitals. The severity or clinical condition of COVID-19 patients was classified into pneumonia, severe pneumonia, ARDS, sepsis, or septic shock according to the WHO guideline [5]. In our analysis, we defined the patients with pneumonia as mild cases and patients with severe pneumonia, ARDS, sepsis, or septic shock as severe cases. Nineteen COVID-19 patients presented with severe cases on admission were excluded. Of the remaining 124 patients, pneumonia progressed to severe cases in 16 patients while ongoing mild diseases (for at least 2 weeks) were reported in 69 patients as of Feb 6. Thirty-nine patients were excluded from this study because of the short duration of follow-up (less than 2 weeks). Finally, we enrolled 85 COVID-19 patients in this study. Patients with or without progression to severe cases by Feb 6 were divided in the progression group or the stable mild group, respectively (Fig. 1). All enrolled patients were followed up until April 1.

### Data collection

The medical records of patients were analyzed by the research team of Huashan Hospital. We obtained epidemiological, demographic, clinical, laboratory, and radiology data from patients’ medical records. The data were reviewed by a trained team of physicians. The date of disease onset was defined as the day when the symptom was noticed. Symptoms, vital signs, laboratory values, chest CT scan, and treatment measures during the hospital stay were collected.

### Statistical analysis

Categorical variables were described as frequency rates and percentages, and continuous variables were described using mean (standard deviation, SD) or median (interquartile range, IQR). Continuous variables were compared using *t* tests, and categorical variables were compared using the *χ*^2^ test or the Fisher exact test. The variates with *P* value less than 0.05 were included into multivariate logistic regression model to determine the potential independent factors. All statistical analyses were performed using SPSS (Statistical Package for the Social Sciences) version 22.0 software (SPSS Inc). *P* value less than 0.05 was considered statistically significant.

## Results

As described in the “Methods” section, this nested case-control study enrolled 85 hospitalized patients with confirmed COVID-19, including 69 in the stable mild group and 16 in the progression group (Table 1). The mean age was 46.6 years (SD, 15.0), and 49 (57.6%) were male. The median duration from the first symptom to hospital admission was 5 days (IQR 4–7 days). Of 85 patients, 36 (42.4%) had 1 or more coexisting medical conditions. Hypertension (26 [30.6%]), diabetes (11 [12.9%]), and chronic liver diseases (9 [9.4%]) were the most common coexisting conditions. None reported chronic lung diseases, malignant tumor, and chronic renal diseases in this cohort. The most common symptom before admission was fever (71 [83.5%]), followed by dry cough (47 [56.0%]), fatigue (39 [45.9%]), and expectoration (29 [34.9%]). Dyspnea (10 [11.8%]) was less seen in the patients because all enrolled patients were mild at admission. Also, there were 10 (11.9%) patients initially presented with diarrhea. Two cases (2.4%) reported to be asymptomatic at admission. Radiology abnormalities were observed in 81 (95.9%) patients, 73 of whom showing bilateral pneumonia. CT scan or X-ray was characterized by multiple peripheral ground-glass opacities.

**Table 1:** Baseline characteristic of study population

	All patients (***n*** = 85)	Stable mild group (***n*** = 69)	Progression group (***n*** = 16)	***P*** value
**Age, years**				< 0.001
Mean ± SD	46.6 ± 15.0	43.9 ± 14.0	58.2 ± 14.0	
Median, range	46 (15–81)	45 (15–80)	62 (30–81)	
**Sex, Male**	49 (57.6)	35 (50.7)	14 (87.5)	0.010
**Symptoms**				
Fever	71 (83.5)	57 (82.5)	14 (87.5)	0.635
Cough	47 (56.0)	38 (55.1)	9 (60.0)	0.728
Expectoration	29 (34.9)	22 (32.4)	7 (46.7)	0.293
Fatigue	39 (45.9)	33 (47.8)	6 (37.6)	0.455
Dyspnea	10 (11.8)	5 (7.2)	5 (31.3)	0.018
Diarrhea	10 (11.9)	9 (13.2)	1 (6.3)	0.679
Headache	5 (6.0)	4 (5.9)	1 (6.3)	1.000
**Laboratory examination**				
White blood count	4.8 ± 1.9	4.8 ± 1.8	5.1 ± 2.2	0.602
Neutrophils	3.2 ± 1.6	3.1 ± 1.6	3.5 ± 1.5	0.380
Lymphocytes	1.2 ± 0.7	1.2 ± 0.6	1.1 ± 1.2	0.418
Hemoglobin	135.7 ± 13.8	136.6 ± 13.6	132.3 ± 14.6	0.272
Platelets	184.6 ± 68.1	190.2 ± 73.0	160.5 ± 33.0	0.117
ALT	30.0 ± 68.1	29.0 ± 19.5	38.0 ± 14.1	0.517
AST	31.3 ± 18.8	29.7 ± 19.5	38.0 ± 14.1	0.114
Creatine	69.2 ± 22.7	64.8 ± 14.9	87.9 ± 37.8	0.029
Creatine kinase	150.4 ± 236.9	136.1 ± 241.7	212.2 ± 211.0	0.250
Troponin T	0.030 ± 0.309	0.024 ± 0.017	0.053 ± 0.054	0.052
Lactate dehydrogenase	240.1 ± 84.3	222.4 ± 73.8	316.4 ± 86.4	< 0.001
NT-proBNP	85.7 ± 200.0	61.7 ± 79.3	189.6 ± 423.5	0.247
C-reactive protein	23.6 ± 25.7	18.1 ± 20.2	47.0 ± 33.7	0.004
**Radiology manifestation**				0.528
Normal	4 (4.7)	4 (5.8)	0 (0%)	
Unilateral involved	8 (9.4)	7 (10.1)	1 (6.3)	
Bilateral involved	73 (85.9)	58 (84.1)	15 (93.5)	
**Chronic medical illness**				
Hypertension	26 (30.6)	17 (24.6)	9 (56.3)	0.032
Coronary heart disease	1 (1.2)	1 (1.4)	0 (0%)	1.000
Diabetes mellitus	11 (12.9)	9 (13.0)	2 (12.5)	1.000
Autoimmune disorders	1 (1.2)	1 (1.4)	0 (0%)	1.000
Chronic liver diseases	9 (9.4)	8 (11.6)	0 (0%)	0.174

Data are shown as *n* (%) or mean ± SD unless specified otherwise

*Abbreviations*: *SD* standard deviation, *ALT* alanine aminotransferase, *AST* aspartate aminotransferase, *NT-proBNP* N-terminal pro-B-type natriuretic peptide

In our cohort, pneumonia progressed in 16 patients. The median time from the onset of illness to severe pneumonia was 8.5 days (IQR, 4.25–10.75 days). When comparing the characteristics on admission between two groups (Table 1), we found that patients in the progression group were more likely to be older (58.2 ± 14.0 vs. 43.9 ± 14.0, *p* < 0.001). Other potential risk factors included being males (87.5% vs. 50.7%, *p* = 0.010), presence of dyspnea (13.2% vs. 6.3%, *p* = 0.018), hypertension (56.3% vs. 24.6%, *p* = 0.032), the higher level of lactase dehydrogenase (LDH) (316.4 ± 86.4 vs. 222.4 ± 73.8, *p* < 0.001), and C-reactive protein (47.0 vs. 18.1, *p* = 0.004). Radiology manifestation was not significantly associated with poor outcome.

Upon adjustment for potential confounding factors with the use of multivariate logistic regression analysis, two independent factors were associated with disease progression: advanced age (odds ratio [OR], 1.012; 95% confidence interval [CI], 1.020–1.166; *P* = 0.011) and the higher level of LDH (OR, 1.012; 95% CI, 1.001–1.024; *P* = 0.038) (Table 2).

**Table 2:** Multivariate logistic regression analysis of potential factors for disease progression

Covariate	Odds ratio	95% CI	***P*** value
Age	1.090	1.020–1.166	0.011
Female	0.113	0.014–1.571	0.113
Hypertension	0.212	0.521–18.884	0.212
Dyspnea	2.319	0.268–20.067	0.445
Creatine	1.032	0.977–1.090	0.264
Lactate dehydrogenase	1.012	1.001–1.024	0.038
C-reactive protein	1.012	0.979–1.046	0.494

*Abbreviation*: *CI* confidential interval

**Fig. 2:**
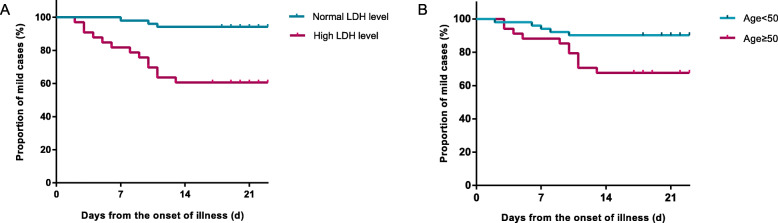
Kaplan-Meier curves for diseases progression according to levels of LDH (**a**) and age (**b**). **a** Patients with LDH above the normal range were at significantly high risk of disease progress than those with normal levels of LDH (hazard ratio [HR], 8.31; 95% CI, 2.96–23.3, *P* < 0.001). **b** Patients aged 50 or older were at increased risk than patients whose age were younger than 50 (HR, 3.56; 95% CI, 1.35–10.2; *P* = 0.011). Abbreviation: LDH, lactase dehydrogenase

Further, patients were stratified by the level of LDH and age. Compared with patients who had normal levels of LDH at admission, those with LDH above the normal range were at significantly high risk of disease progress (hazard ratio [HR], 8.31; 95% CI, 2.96–23.3, *P* < 0.001) (Fig. 2a). Besides, patients aged 50 or older were at increased risk as well (HR, 3.56; 95% CI, 1.35–10.2; *P* = 0.011) (Fig. 2b). As of April 1, all patients in stable mild group and 68.7% (11/16) in the progression group were cured and discharged; the remaining 5 patients in the progression group were still hospitalizing in the ICU.

## Discussion

This is the first known analysis to focus on identifying high-risk COVID-19 patients for developing severe illness. To identify a small portion of high-risk patients among the whole population of mild patients, we excluded those who presented with severe illness on admission, thus only comparing patients with stable mild illness and those who deteriorated from mild to severe illness.

The initial symptoms and signs in COVID-19 patients were usually very mild. However, as the disease progresses, some patients’ symptoms would deteriorate. According to public data of China CDC and previous studies, about 8–30% of patients would eventually develop severe illness and about 1–11% of patients would die [3]. Guan et al. [6] reported that 40% of ICU patients were non-severe on admission. With proactive screening of close contacts and quicker diagnostic procedures, this proportion may be even higher. Comparing the mild cases with or without deterioration will help clinicians identifying potentially critical patients earlier, allocating medical resources more reasonably, paying more attention to these patients, thus giving necessary interventions as early as possible. Previous studies have uncovered that there were numerous disparities in background illness, vital signs, and laboratory parameters between mild and severe patients, including lymphocytes, prothrombin time, creatine kinase, LDH, and so on [3, 6, 7]. However, which parameter dynamic change that initiates earlier remains unanswered.

LDH is a cytoplasmic glycolytic enzyme found in almost every tissue. Its elevation generally indicates tissue damage. Raised LDH was a common findings in patients infected with MERS-CoV [8–10], H7N9 [11, 12], and H5N1 [13]. It was reported to be an independent factor of mortality for patients with severe acute respiratory syndrome [14] and H1N1 infection [15]. It was also one of the biomarkers most strongly associated with ARDS mortality [16, 17]. Our finding of increased LDH in the early phase of severe COVID-19 cases suggested possible subclinical tissue damage. Although the virus binds to human angiotensin converting enzyme 2 (ACE2) receptor in the lung [18, 19], which explains why the lungs are the first organs affected, but as the disease progresses, various cytokine abnormalities and multiple organs dysfunction can be found in severe patients [3, 7], indicating systemic organ damage caused by the excessive activation of the immune system. LDH isoenzymes test can further help to locate damaged tissues or organs.

In this study, the comparison between the two groups showed differences in gender, history of hypertension, dyspnea symptom, creatinine, and C-reactive protein levels. These systemic factors also support that the pathophysiology of critically ill patients might be the systemic activation of immune response. History of hypertension was relatively rare as a factor in disease progression for an infectious disease; however, it was widely reported to be associated with disease severity of COVID-2019 [6, 7]. This was thought to be related to the virus binding receptor. Structural analysis suggested that SARS-CoV-2 might be able to bind to the ACE2 receptor [18, 19], which shared some homology with ACE and played a role in the renin-angiotensin system. The influence of blood pressure on prognosis may be related to this, but the specific mechanism remains unknown.

This study had several limitations. First, we did not measure viral load and some patients lacked coagulation function testing, which could be factors related to the severity of the disease. Second, we did not test the LDH isoenzymes due to limited resources. LDH isoenzyme analysis in the future may help to identify the source of increased LDH. Third, the sample size of our study is relatively small, but the multicenter and prospective nature of our study should reduce the bias and increase the generalizability.

## Conclusions

In this multicenter nested case-control study, advanced age and high LDH level were independent risk factors for deterioration in mild COVID-19 patients. Among the mild patients, clinicians should pay more attention to the elderly patients or those with high LDH levels.

## Data Availability

The data that support the findings of this study are available from the corresponding author on reasonable request.

## Abbreviations

ARDS: Acute respiratory distress syndrome
COVID-19: Coronavirus disease 2019
HR: Hazard ratio
IQR: Interquartile range
LDH: Lactase dehydrogenase
OR: Odds ratio
SD: Standard deviation
